# A Comparative Study of Poly(Azure A) Film-Modified Disposable Electrodes for Electrocatalytic Oxidation of H_2_O_2_: Effect of Doping Anion

**DOI:** 10.3390/polym10010048

**Published:** 2018-01-06

**Authors:** Jerónimo Agrisuelas, María-Isabel González-Sánchez, Beatriz Gómez-Monedero, Edelmira Valero

**Affiliations:** Department of Physical Chemistry, School of Industrial Engineers, University of Castilla-La Mancha, Campus Universitario s/n, 02071 Albacete, Spain; Jeronimo.Agrisuelas@uv.es (J.A.); MIsabel.Gonzalez@uclm.es (M.-I.G.-S.); Beatriz.Gomez@uclm.es (B.G.-M.)

**Keywords:** conducting polymers, poly(azure A), sodium dodecyl sulfate, electrochemical sensor, disposable screen-printed electrodes, hydrogen peroxide

## Abstract

In the present paper, poly(azure A) (PAA) films were electrosynthetized in the presence of different doping anions on disposable screen-printed carbon electrodes (SPCEs). The anions used included inorganic monoatomic (chloride and fluoride), inorganic polyatomic (nitrate and sulfate) and organic polyatomic (dodecyl sulfate, DS) species. The coated electrodes thus obtained were characterized by electrochemical techniques and SEM. They showed improved electrocatalytic activities towards hydrogen peroxide oxidation compared to that of a bare SPCE. In particular, the insertion of DS anions inside PAA films provided a special sensitivity to the electrocatalysis of H_2_O_2_, which endowed these electrodes with promising analytical features for H_2_O_2_ quantification. We obtained a wide linear response for H_2_O_2_ within a range of 5 µM to 3 mM and a limit of detection of 1.43 ± 0.10 µM (signal-to-noise ratio of 3). Furthermore, sensitivity was 72.4 ± 0.49 nA·µM^−1^∙cm^−2^ at a relatively low electrocatalytic oxidation overpotential of 0.5 V vs. Ag. The applicability of this boosted system was tested by the analysis of H_2_O_2_ in commercial samples of a hair lightener and an antiseptic and was corroborated by spectrophotometric methods.

## 1. Introduction

Hydrogen peroxide has been traditionally electrocatalyzed and detected using platinum-based electrodes [1,2,3] since this metal is a good catalyst for H_2_O_2_ decomposition. However, due to its high cost, recycling platinum from waste screen-printed electrodes for the development of new sensors has recently been proposed [4]. The oxidation or reduction of H_2_O_2_ on other typical electrodes can be limited by a slow electron transfer rate and high over potentials [5]. Moreover, enzyme-based electrochemical biosensors have a relatively high cost and unstable activities [6]. For these reasons, the search of new materials to solve these shortcomings while presenting similar electroanalytical properties is of paramount importance.

Conducting polymers (CPs) are an appealing approach for electrocatalytic applications given their special characteristics, such as cost-effectiveness, facile synthesis, stability, reproducibility and good sensitivity [7,8,9,10]. The electrochemical and conducting properties of CPs depend largely on the electrosynthesis procedure [11,12,13,14]. During this process, the polymeric structure and doping agents are fixed in CPs, which confer their special characteristics. Among doping agents, the insertion of electrolytes seems to be one of the most cost-effective options [15].

Among the several strategies developed to reduce costs in the field of electroanalysis, screen-printing is a suitable and simple technique for the mass production of disposable electrodes and design of (bio)sensors [16]. Nowadays, the use of screen-printed electrodes (SPEs) is increasingly extending since they combine several advantages, such as versatility, cost-effective manufacture, minimum analysis volume while avoiding the tedious polishing of solid electrodes and offering the possibility of in situ analysis [17,18,19,20]. As far as we know, disposable SPEs modified with organic CPs are still commercially limited, although a number of companies can offer customized solutions.

The electropolymerization of azines and derivatives (e.g., neutral red, azure A or methylene blue) provides an important class of CPs with numerous applications in the sensors field [3,21]. The resulting polymer has two kinds of electroactive double bonds: one between two adjacent monomer molecules and the other within the heterocyclic ring with the participation, in both, of anions and protons for the charge balance [22,23]. Nonetheless, research on SPEs modified by azine-derivative polymers is scarce [24,25,26,27].

As a more economical alternative to Pt electrodes, the aim of the present paper was to compare the electroactivity and electrocatalytic oxidation of H_2_O_2_ driven by disposable screen-printed carbon electrodes (SPCEs) modified by Azure A polymers (PAA). Several PAA films were electrosynthetized under identical experimental conditions, but in different electrolyte solutions including inorganic monoatomic anions (chloride and fluoride), inorganic polyatomic anions (nitrate and sulfate), and an organic polyatomic anion (dodecyl sulfate, DS). The data obtained and their interpretation will be of great interest for extending the use of this type of polymers on disposable electrodes and improving the electrosynthesis of noteworthy films on SPCEs for future sensing applications.

## 2. Materials and Methods

### 2.1. Chemicals and Solutions

l-Ascorbic acid (sodium salt), azure A (80%), caffeine, citric acid (trisodium salt), l-dehydroascorbic acid (DHA), d(+)-glucose, H_2_O_2_ (35%), K_2_SO_4_ (99%), Ru(NH_3_)_6_Cl_3_ (98%) and sodium dodecyl sulfate (SDS, 95%) were purchased from Sigma-Aldrich (Madrid, Spain). Reagents for the spectrophotometric measurement of H_2_O_2_ by the classical xylenol orange method (ammonium iron (II) sulfate hexahydrate, d(−)-sorbitol and xylenol orange (disodium salt)) [28] were also acquired from Sigma-Aldrich. KF and KNO_3_ (99.5%) were obtained from Fluka (Darmstadt, Germany). KCl (99.5%) and ethanol (99.9%) were purchased from Scharlau (Barcelona, Spain). KH_2_PO_4_ (99.5%) and K_2_HPO_4_ (99%) were sourced from Merck (Darmstadt, Germany). Potassium ferrocyanide (99.95%) was obtained from Probus (Badalona, Spain) and H_2_SO_4_ (95–98%) was obtained from Panreac (Castellar del Vallès, Spain). The samples of hair lightener (stated composition: water, alcohol denat, Chamomilla recutita flower extract, hydrogen peroxide, parfum, phosphoric acid, amyl cinnamal, coumarin, linalool) and antiseptic (3% hydrogen peroxide) were purchased from a local supermarket. All the reagents were used as received with no further purification. Stock solutions of samples were prepared by appropriate dilutions in 0.1 M potassium phosphate buffer (pH 7).

Solutions were prepared with demineralized water purified by a Milli-Q purification system (18.2 MΩ·cm) (Millipore Corp, Bedford, MA, USA). For the polymerization solutions, 0.02 M of the appropriate electrolyte was prepared in aqueous solution. Subsequently, 0.1 M phosphate buffer solution (pH 7) was prepared from K_2_HPO_4_ and KH_2_PO_4_ to be used as the supporting electrolyte for electrochemical measurements.

### 2.2. Electrosynthesis of PAA Films

PAA films were electrogenerated on the surface of the working electrode (carbon) of disposable SPCEs (DRP-150, DropSens, Oviedo, Spain) with an AUTOLAB potentiostat-galvanostat set-up (PGSTAT204) controlled by the NOVA 2.0 software package. The geometrical area of these electrodes was 12.6 mm^2^, which was used to calculate current densities. A Pt foil (area = 0.49 cm^2^) was used as the counter electrode during PAA electrosynthesis to keep the counter electrode integrity of the SPCE. The potentials used herein were consistently based on the Ag pseudo-reference electrode of SPCEs.

The electrosynthesis solution consisted of a dissolution of 1 mg·mL^−1^ of azure A in 0.02 M either KCl, KF, KNO_3_, K_2_SO_4_ or SDS. To this end, twenty voltammetry cycles were carried out between −0.25 V and 1 V at 10 mV·s^−1^ (initial potential, *E_i_* = 0.5 V). The modified disposable electrodes thus obtained were named as PAA(anion used in electrosynthesis), i.e., PAA(Cl), PAA(F), PAA(NO_3_), PAA(SO_4_) and PAA(DS). After deposition, the modified SPCEs were rinsed with abundant ethanol to remove the residual monomers adsorbed on PAA films. They were subsequently cleaned with abundant double-distilled water to eliminate residual ethanol. Finally, dry electrodes were stored in airtight containers while not in use.

Modified SPCEs were characterized by cyclic voltammetry in 0.1 M phosphate buffer. For this purpose, the Pt and Ag electrodes from SPCEs were used as counter and reference electrodes, respectively. Cyclic voltammetries were performed from 0.5 V to −0.7 V, and vice versa, unless otherwise specified.

### 2.3. Electrochemical Impedance Spectroscopy (EIS)

EIS of the modified SPCEs was carried out at 0.12 V in 5 mM potassium ferrocyanide and 0.1 M KCl aqueous solution, by means of an AUTOLAB potentiostat-galvanostat set-up (PGSTAT128N) equipped with a frequency response analyzer (FRA) module. Modified working electrodes were polarized for 60 s. A sinusoidal small amplitude potential perturbation (5 mV *rms*) was subsequently superimposed between 65 kHz and 10 mHz, with five points per decade. The experimental data were fitted to the equivalent circuit by means of the EIS Spectrum Analyzer (v 1.0, Physico-Chemical Research Institute, Belarusian State University, Minsk, Republic of Belarus) [29].

### 2.4. Electrocatalytic Oxidation of H_2_O_2_

Cyclic voltammetry and amperometry were used to compare the electrocatalytic capabilities towards H_2_O_2_ oxidation of the PAA films obtained in 0.1 M phosphate buffer (pH 7). Buffered solutions (10 mL) remained under constant magnetic agitation at room temperature. Calibration was obtained by successive H_2_O_2_ additions and by measuring the current intensity after stabilization.

### 2.5. Scanning Electron Microscopy Images

The surface morphology of all the PAA films electrosynthetized herein was examined by scanning electron microscope (SEM) (mod. Jeol LTD., JSM-6469LV, Akishima, Japan) at an acceleration voltage of 20 kV. It was necessary to cover samples with a gold-platinum thin film of about 2 nm by sputtering to avoid any electric charge effect, which could affect image acquisition. A K-575X Emitech Sputter Coater from Quorum Technologies (Quorum Technologies Ltd., East Grinstead, West Sussex, UK) was used for this purpose.

### 2.6. Spectroscopy

Spectrophotometric measurements were taken in a UV/Vis Perkin-Elmer Lambda 35 (Perkin Elmer Instruments, Waltham, MA, USA) spectrophotometer. Hydrogen peroxide concentration in the samples was measured by the conventional xylenol orange method at 550 nm following the instructions given by the supplier.

## 3. Results and Discussion

### 3.1. Electrosynthesis of PAA Films on SPCEs

Doping ions used in the synthesis solution as the counter-charge to maintain the electroneutrality principle during macromolecular structure formation of CPs define the size and shape of the hydrated cavities inside polymeric films and, as a result, strongly affect their electrochemical properties [30,31,32]. As PAA films are unresponsive to the cation in the electrosynthesis solution [27], we focused on the effect of three kinds of anions in the electrosynthesis solution, namely: (i) inorganic monoatomic anions, e.g., chloride and fluoride; (ii) inorganic polyatomic anions, e.g., nitrate and sulfate; and (iii) one organic polyatomic anion, dodecyl sulfate.

**Figure 1:**
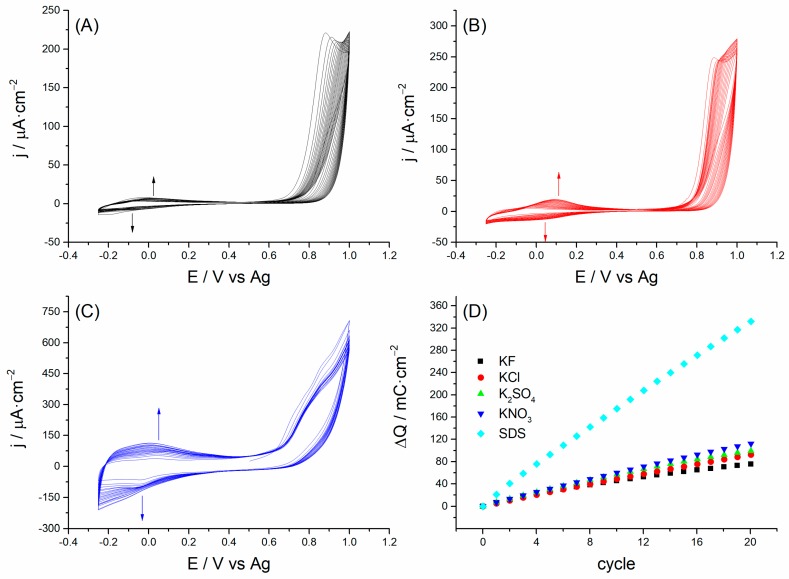
Polarization curves of 1 mg·mL^−1^ azure A in 0.02 M KF (**A**), K_2_SO_4_ (**B**) and SDS (**C**) aqueous solutions at a scan rate of 10 mV·s^−1^ between −0.25 V and 1 V. The voltammetry cycle starts at 0.5 V. (**D**) Charge accumulation during the electrochemical reactions of PAA(anion) electrosynthesis calculated from the voltammetric cycles shown in (**A**–**C**) and Figure S1.

Figure 1 shows the voltammetric response of PAA films electrodeposited on the surface of SPCEs using 1 mg·mL^−1^ of azure A in 0.02 M KF (Figure 1A), K_2_SO_4_ (Figure 1B) and SDS aqueous solution (Figure 1C). The electrosynthesis of PAA, using either 0.02 M KCl or KNO_3_ showed very similar profiles to the results observed for KF or K_2_SO_4_, respectively (data in Figure S1 of the Supplementary Material). In all the electrosynthesis solutions, azure A was radicalized by electrochemical oxidation above 0.5 V [33]. Radical cations thus formed connections to one other by creating stable covalent bonds between monomers (amine-based intermonomeric links). Polymerization progress was evidenced by the increase in current observed between 0.5 V and −0.25 V, which is directly related with the amount of amine-based intermonomeric links formed during the polymerization process [34]. As seen in Figure 1, the achieved voltammetric currents increased as the anions in solution became molecularly more complex.

Integrating these currents over the experimental time allowed us to calculate the consumed charge during the electrode reactions, which is proportional to the amount of electroactive PAA deposited on SPCEs. Figure 1D shows the accumulated charge after each voltammetric cycle for the electrosynthesis of the different PAA films. Two different kinds of evolution of charge can be distinguished.

On the one hand, for both inorganic monoatomic and polyatomic anions, the charge increase was almost linear up to the tenth cycle and then curved. The formation of two layers with a different morphology and structure in the electrosynthetized CP might be the reason [35]. The internal layer in contact with the surface electrode would be the electroactive part of film, while the external layer in contact with the bulk solution would act as a diffusion membrane that hinders the ionic exchange between the electroactive layer and the solution.

On the other hand, the charge consumed during PAA electrosynthesis in SDS solutions was significantly the highest one and maintained a near linear increase, at least during the first 20 cycles. In this case, SDS would favor the polymerization process and might be responsible for this more pronounced linear increase since this anionic surfactant concentrates azure A monomers into SDS micelles [36], thus enhancing the electron transfer between the surface of the PAA film and the monomers in solution [32,37,38].

### 3.2. Electrochemical Response

**Figure 2:**
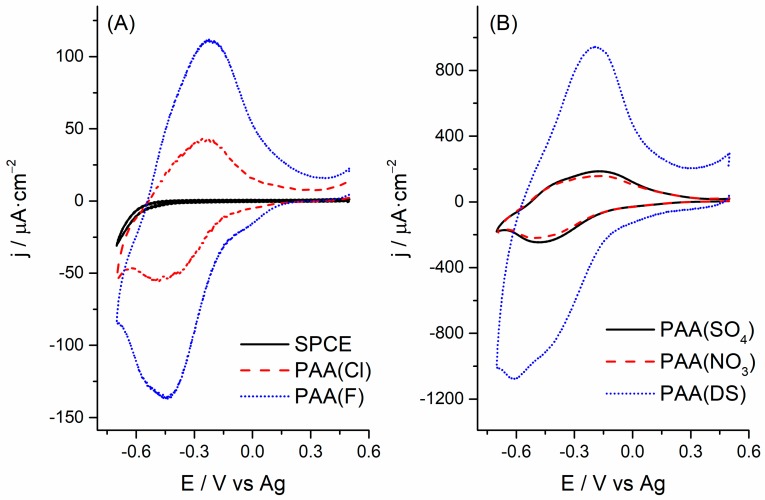
Cyclic voltammograms obtained at a bare SPCE (**A**) and the modified SPCEs with the different PAA films (PAA(Cl) and PAA(F) in (**A**), and PAA(SO_4_), PAA(NO_3_) and PAA(DS) in (**B**) at a scan rate of 50 mV·s^−1^ in 0.1 M phosphate buffer (pH 7).

Figure 2 shows the electrochemical response of a bare SPCE and the synthetized PAA films in 0.1 M phosphate buffer (pH 7). As can be observed, the bare SPCE showed no relevant electroactivity around −0.3 V (Figure 2A, black line). In contrast, PAA films exhibited good electroactivity as revealed by a well-defined pair of electrochemical peaks (Figure 2A,B). Notably, the charge consumed during a cyclic voltammogram of PAA(DS) films (Figure 2B, blue line) was between two-fold and five-fold higher compared to the charge consumed for the other PAA(anion) films. These results corroborated the findings mentioned in the preceding paragraph, and indicate that PAA(DS) films on SPCEs have more electroactive sites than the other films.

### 3.3. Surface Characterization

**Figure 3:**
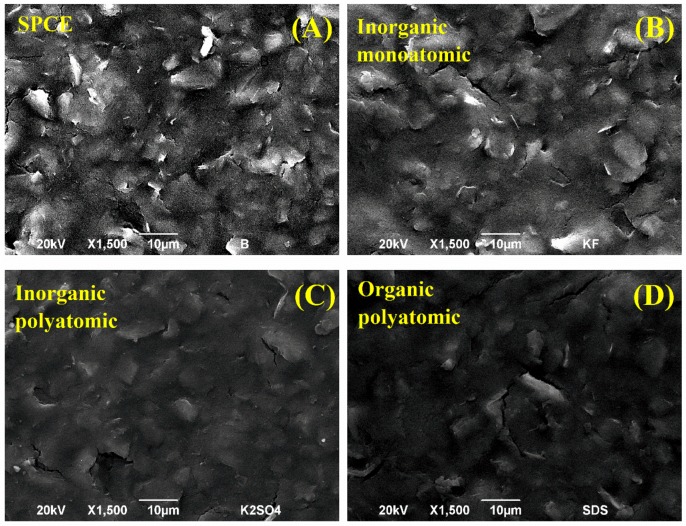
SEM images of (**A**) a bare SPCE and some modified SPCEs by: (**B**) PAA(F), (**C**) PAA(SO_4_) and (**D**) PAA(DS) films. All the images were recorded at 1500× magnification and markers correspond to 10 µm.

The electrode surface of the PAA modified SPCEs was characterized by SEM (Figure 3). The surface of a bare SPCE (Figure 3A) shows a similar roughness to SPCEs modified by PAA films electrosynthetized with inorganic monoatomic anions (Figure 3B). As the anion used during the electrosynthesis was more complex, the electrode surface seemed smoother since a larger amount of PAA was deposited. SPCEs modified by PAA films electrosynthetized with inorganic polyatomic anions (Figure 3C) and with organic polyatomic anions (Figure 3D) significantly covered the working electrode of the SPCE. This tendency is consistent with our discussion on the information extracted from Figure 2.

### 3.4. Electrochemical Impedance Spectroscopy

**Figure 4:**
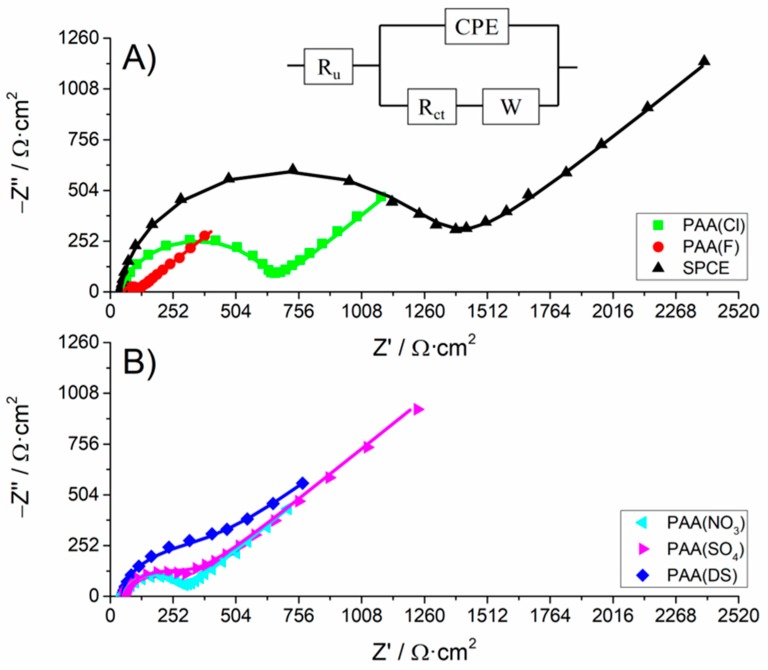
Nyquist plots of a bare SPCE (A), black line) and the different PAA(anion) films-modified SPCEs herein prepared, polarized at 0.12 V, measured in 5 mM potassium ferrocyanide and 0.1 M KCl aqueous solution. The perturbation frequency range was from 65 KHz to 10 mHz with an amplitude of 5 mV *rms.* The stabilization time was 60 s. The inset shows the equivalent circuit where *R_s_* is the uncompensated resistance, *R_ct_* is the charge transfer resistance, CPE is the constant-phase element and *W* is the Warburg impedance. The points represent experimental data and the lines correspond to the fittings.

Frequency dependent impedance provides the finest information to characterize the surface of electrodes modified with electroactive polymers within the potential range of electroactivity. Figure 4 shows the typical Nyquist plots obtained for a bare SPCE and the SPCEs modified with the different PAA(anion) films in the presence of a redox probe. On the one hand, the electron transfer rate of the ferrocyanide at the electrode|solution interface defines the size of the semicircle or arc portion in the EIS spectrum at relatively high frequencies. On the other hand, the diffusional limiting step of the electrochemical process is shown as the linear portion with a slope of 45° at relatively low frequencies.

Data were fitted to a standard Randel’s equivalent circuit depicted in the inset of Figure 4. The uncompensated resistance ($R_{s}$) describes the electrolyte resistance. The charge transfer resistance ($R_{ct}$) depends on the dielectric and insulating features at the electrode and electrolyte interface. The Warburg impedance (W) represents the bulk properties of the electrolyte solution and diffusion features of the redox probe in solution at the lower frequencies. The double layer capacitance was characterized by a constant-phase element (CPE). As observed in Figure 4, a good agreement between the circuit model and the measurement system was obtained. Table 1 shows that the PAA modified SPCEs and the bare SPCE have similar $R_{s}$ around 40–50 Ω·cm^2^. CPE increased as the PAA amount on the electrode increased and CPE exponent (α) was very close to 1 in all electrodes, indicating a highly smooth and homogenous electrode surface in accordance with SEM images (Figure 3) [39,40]. The variation of the value of the Warburg impedance suggested that the diffusion process depended on the singular steric hindrance for this process in each PAA film [41].

Finally, $R_{ct}$ values clearly proved that PAA films significantly reduced $R_{ct}$ relative to $R_{ct}$ of a bare SPCE. It is worth noting the $R_{ct}$ obtained for the PAA(F) film (around 49 Ω·cm^2^) and for the PAA(DS) film (around 292 Ω·cm^2^). In the former case, an electrochemical fluorination of PAA cannot be discarded during the PAA electrosynthesis in KF solutions [42], endowing these films with unexpected properties. The functionalization of PAA films could be an interesting line for further research. In the latter case, it is important to note that $R_{ct}$ for PAA(DS) was close to other PAA films (PAA(NO_3_) or PAA(SO_4_)), despite PAA(DS) being considered the thickest film in light of the results previously described. This means that the conducting properties of CPs are improved by SDS compared to CPs electrosynthetized in traditional solutions [27,43].

### 3.5. Electrocatalytic Activity

**Figure 5:**
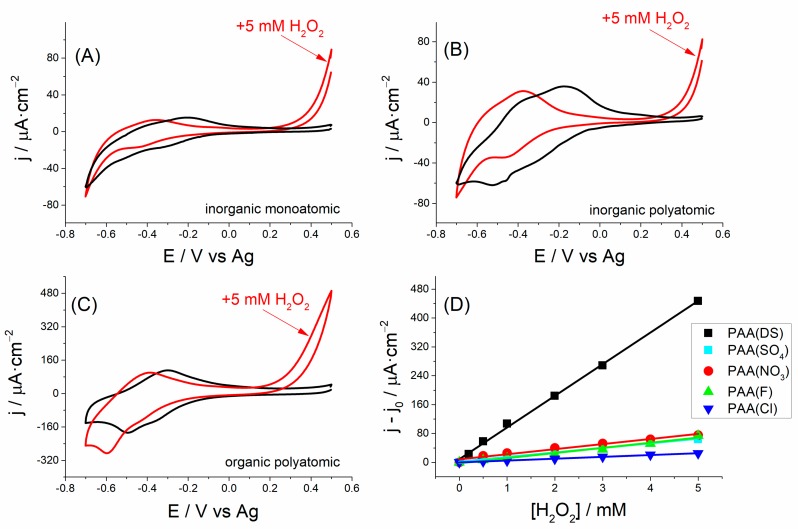
Cyclic voltammograms (scan rate: 10 mV·s^−1^, 0.1 M phosphate buffer (pH 7)) of different PAA(anion) films in the absence (black lines) and presence of 5 mM H_2_O_2_ (red lines) for PAA(F), PAA(SO_4_) and PAA(DS) films ((**A**–**C**), respectively). (**D**) Current at 0.5 V vs. H_2_O_2_ concentration for all modified SPCEs. Error bars correspond to three replicates for each modified electrode.

PAA films or derivatives have proved to be good detectors for H_2_O_2_ [44,45]. In this work, the electrocatalytic oxidation of H_2_O_2_ driven by the PAA films synthesized in the presence of a variety of anions on SPCEs was initially investigated by cyclic voltammetry. Figure 5 shows the cyclic voltammograms obtained in the absence and the presence of hydrogen peroxide for PAA(F) (Figure 5A), PAA(SO_4_) (Figure 5B) and PAA(DS) (Figure 5C). As expected in this kind of films which act as redox mediators [44], H_2_O_2_ addition caused the cathodic peak of the PAA(anion) films to increase, while the anodic peak decreased, and in both cases with a significant shift in the respective peak potentials to more cathodic potentials. Simultaneously, a large increment in current at around 0.5 V was also observed. In recent studies [46], the electrocatalytic oxidation of H_2_O_2_ by poly(*N*-methylthionine) (or poly(azure C)) has been attributed to the formation of radicals on the electroactive heterocyclic nitrogen atoms in the phenothiazine ring enhancing the electron transfer between the electrode and H_2_O_2_. A similar mechanism may be extrapolated to PAA films because the corresponding monomeric structures of azure A and azure C only differ in a methyl group.

In our case, the initial state of PAA was assumed completely deprotonated at pH 7 [27]. Then, the polymer would be oxidized to a free radical (Equation (1)) when applied potentials arrived to 0.5 V. Free radicals in phenazine derivatives have been assumed to form in some conditions [47,48]. Cationic radicals would immediately be reduced by H_2_O_2_, yielding again the initial state of PAA, and ${\mathrm{HO}}_{2}^{●}$ (Equation (2)). The ${\mathrm{HO}}_{2}^{●}$ free radical would end decomposing into O_2_ and H^+^ (Equation (3)).

(1) $$\mathrm{PAA}\to {\mathrm{PAA}}^{●+}+e^{-}$$

(2) $${\mathrm{PAA}}^{●+}+H_{2}O_{2}\to \mathrm{PAA}+{\mathrm{HO}}_{2}^{●}+e^{+}$$

(3) $${\mathrm{HO}}_{2}^{●}\to O_{2}+H^{+}+e^{-}$$

Focusing our attention around 0.5 V, we observed that the current increment on this zone yielded a greater sensitivity than the cathodic peak current (around −0.5 V) in all modified SPCEs. In particular, we observed that the polymer synthesized in SDS showed the maximum current increase (Figure 5C). In a detailed calibration plot with the addition of different H_2_O_2_ concentrations (Figure 5D), PAA(DS) films showed the best sensitivity among all modified SPCEs.

**Figure 6:**
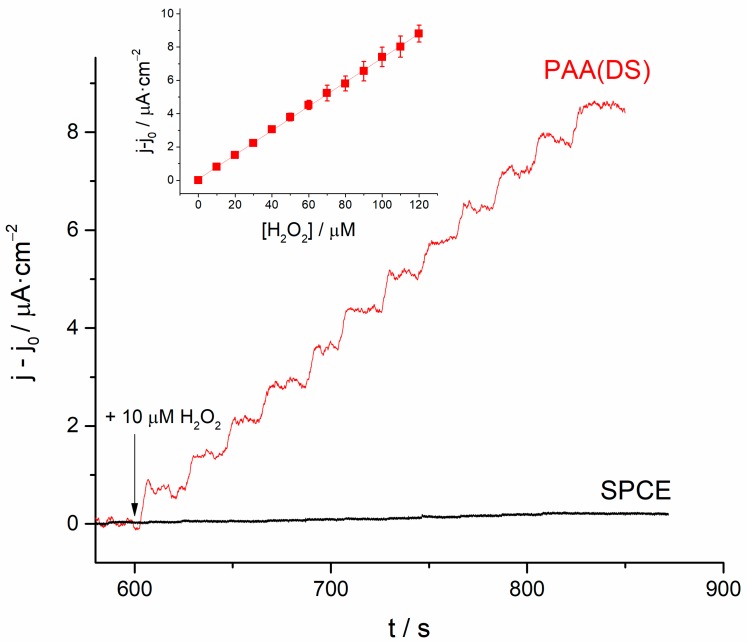
Amperometric response of a bare SPCE (black line) and a PAA(DS)|SPCE (red line) upon successive additions of 10 µM H_2_O_2_ in phosphate buffer solution (pH 7) at 0.5 V. The inset shows the calibration straight line obtained from the current for the PAA(DS)|SPCE. Error bars are related to the standard deviation of three different measurements.

Based on the best sensing performance obtained at 0.5 V, we tested the potential application of the PAA(DS) films deposited on SPCEs to measure H_2_O_2_ by amperometry. The different H_2_O_2_ concentrations in 0.1 M phosphate buffer (pH 7) were measured on a bare SPCE and a PAA(DS) modified SPCE after polarization of the electrodes at 0.5 V for 600 s. Figure 6 shows the amperometric response to successive additions of H_2_O_2_ for both electrodes. As seen, the bare SPCE showed a poor current signal response to H_2_O_2_ additions. Conversely, the PAA(DS) modified SPCE showed a well-defined step response. The current generated was used to calculate the sensitivity and limit of detection (LOD) of the system (inset of Figure 6). Sensitivity was calculated to be 72.4 ± 0.49 nA·µM^−1^∙cm^−2^ and the LOD was 1.43 ± 0.10 µM (estimated at a signal-to-noise ratio of 3). The amperometric response of the same electrode previously used was once again tested after 160 min, and it was kept constant with no significant loss of signal, which indicated the valuable stability of the modified electrode. Sensor repeatability was high with an RSD of 3.4% (*n* = 5). Reproducibility using three different electrodes was 6.2%. The sensor also demonstrated good stability since after 25 measurements of 10 µM H_2_O_2_ it maintained 100% of the initial signal. Additionally, it should be mentioned that the linear range was notably wide, from 5 µM to 3 mM (see Figure S2 in the Supplementary Material).

The analytical parameters obtained with our system fell within the range of other non-enzymatic sensors of H_2_O_2_ based on azine derivative polymers, which reached LODs of a micromolar order. Table 2 shows a comparative study of the analytical performance for the non-enzymatic sensing of H_2_O_2_ for different modified electrodes with a variety of conjugated polymers [46,49,50,51,52,53,54,55]. As can be seen, sensitivity reached with our system shows an acceptable value, similar to that obtained by other authors. It is important to bear in mind that most modified electrodes shown in Table 2 are based on conventional electrodes, such as glassy carbon (GC). Very few works exist on non-enzymatic hydrogen peroxide sensing using CPs deposited on disposable SPEs. The only sensor using CPs-modified SPEs was in [51], where the authors achieved excellent results, such as good sensitivity and a low LOD. However, the fabrication procedure in this report required a previous chemical step for the synthesis of nanoparticles (NPs), and then the use of piezoelectric inkjet printing techniques to deposit these NPs on the electrode surface. In contrast, the polymerization process to prepare the PAA(DS) modified SPCEs herein proposed is easy and fast, and the only requirement is a very simple electrochemical step using commercial electrodes, which allows the device to be ready for use with no complex manufacturing in just 2 h. Additional advantages of the PAA(DS) modified SPCEs include the excellent benefits of disposable screen-printed electrodes, such as simplicity, low cost, the possibility of using microvolumes and in situ analyses.

In order to test the applicability of this sensor with real samples, interferences from a number of substances were examined. Ethanol, sodium citrate, glucose, caffeine and DHA were added to the cell to obtain final concentrations of 0.1 mM and no response was detected (Figure S3 in Supplementary Material). Furthermore, the signal for 50 µM H_2_O_2_ was the same in both the absence and presence of these substances. However, sodium ascorbate was able to affect the amperometric signal (inset of Figure S3, Supplementary Material). Nevertheless, this interference can be solved by the addition of ascorbate oxidase [56] since the product of the enzymatic process, DHA, does not interfere the signal.

Finally, in order to check the applicability of the PAA(DS) modified SPCEs herein prepared, they were used to determine the H_2_O_2_ concentration in a commercial hair lightener and an antiseptic. The concentrations calculated by the standard additions method using amperometry were compared to those obtained by a standard spectrophotometric method with xylenol orange (Table 3). The results obtained by these two methods were similar, with a coefficient of variation (% CV) of <1%, which revealed the good precision of PAA(DS) films for H_2_O_2_ sensing.

## 4. Conclusions

In this work, PAA films were successfully deposited on disposable SPCEs using different doping anions in the electrosynthesis solution. Of all the anions classified as inorganic monoatomic, inorganic polyatomic and organic polyatomic, dodecyl sulfate anions allowed the synthesis of a PAA film with significantly improved electrochemical properties, despite the film deposited in the presence of dodecyl sulfate substantially covering the working electrode surface. Polymerization in the presence of this surfactant allowed a better electrocatalytic oxidation of H_2_O_2_ compared to the same polymer electrosynthetized in the presence of other anions or a bare SPCE. The applicability of the modified electrode as a hydrogen peroxide sensor has been shown, with a linear response for this molecule within the 5 µM to 3 mM range, a 1.43 ± 0.10 µM limit of detection (signal-to-noise ratio of 3) and 72.4 ± 0.49 nA·µM^−1^∙cm^−2^ sensitivity. In addition, the hydrogen peroxide concentration was measured in real commercial hair lightener and antiseptic samples, with similar results to those obtained by a standard spectrophotometric method. Therefore, the use of PAA(DS) films-modified SPCEs to measure peroxides represents a simple method that avoids long and complex sensor fabrication processes, and one that could be the basis for manufacturing new low-cost sensor electrodes. These results can be extrapolated to the electrodeposition of PAA(DS) films in different kind of electrodes intended for H_2_O_2_ electrocatalysis.

## Figures and Tables

**Table 1:** Values of the equivalent circuit elements obtained by fitting the experimental results shown in Figure 4.

Electrode	$R_{s}$ Ω·cm^2^	CPE μF·cm^−2^	α	$R_{ct}$ Ω·cm^2^	W Ω·cm^2^·s^−0.5^
SPCE	42	33	0.9	1233	279
PAA(Cl)	46	29	0.9	585	115
PAA(F)	56	29	1.0	49	75
PAA(NO_3_)	52	56	0.9	227	110
PAA(SO_4_)	52	140	0.9	254	232
PAA(DS)	50	1900	1.0	292	132

**Table 2:** Comparison of the electroanalytical parameters used for a variety of electrodes modified with different CPs for the non-enzymatic sensing of H_2_O_2_.

Electrode ^a^	E (V)	Reference Electrode	LOD (μM)	Sensitivity (nA·μM^−1^·cm^−2^)	Reference
Pt foil|PNMTh	0.60	SCE	0.1	2786.0	[46]
GCE|PAA-CS|Cu	−0.40	SCE	0.7	88.9	[49]
PAA-PNR|MWCNTs	−0.25	Ag/AgCl	1.0	10.3	[50]
SPCE|PBNPs	0.00	Ag/AgCl ^b^	0.2	762.0	[51]
GCE|PMB-FAD	−0.45	Ag/AgCl	0.1	1109.0	[52]
CF|PEDOT-PNR	−0.36	SCE	80	0.9	[53]
GCE|PTH-Au	−0.10	Ag/AgCl	0.2	14.1	[54]
GCE|PBCB|SWCNT	−0.30	SCE	120.0	58.1	[55]
PAA(DS)|SPCE	0.50	Ag	1.4	72.4	This work

^a^ CF. carbon film, CS: chitosan, FAD: flavin adenine dinucleotide, GCE: glassy carbon electrode, MWCNT: multi-walled carbon nanotube, PBCB: poly(brilliant cresyl blue), PBNP: Prussian blue nanoparticles, PEDOT: poly(3,4-ethylenedioxythiophene), PMB: poly(methylene blue), PNMTh: poly(*N*-methylthione), PNR: poly(neutral red), PTH: poly(thionine), SCE: saturated calomel electrode, SWCNT: single-walled carbon nanotubes. ^b^ This is a pseudo-reference electrode made of conductive ink.

**Table 3:** Hydrogen peroxide found in the commercial samples measured by both the electrochemical and xylenol orange methods.

Sample ^a^	Electrochemical Method (M)	Spectrophotometric Method (M)	Recovery (%)
Hair lightener	1.20 ± 0.01	1.19 ± 0.04	100.8
Antiseptic	0.84 ± 0.02	0.85 ± 0.04	98.9

^a^ The samples were diluted in phosphate buffer (pH 7) to be measured within the linear range of each analytical method. Three replicates were performed. Values are given as mean ± standard deviation.

## Supplementary Materials

The following are available online at www.mdpi.com/2073-4360/10/1/48/s1, Figure S1: Polarization curves of 1 mg·mL^−1^ azure A in 0.02 M KCl (A) and KNO_3_ (B) aqueous solutions at scan rate 10 mV·s^−1^ between −0.25 V and 1 V. The voltammetry cycle stars at 0.5 V; Figure S2: Calibration straight line of the PAA(DS) obtained from the amperometric response of such electrode upon successive additions of H_2_O_2_ in phosphate buffer solution (pH 7) at 0.5 V. The error bars correspond to standard deviations between three replicates using the same electrode; Figure S3: Amperometry of the influence of certain compounds measured at 0.5 V. 50 µM hydrogen peroxide, 100 µM ethanol, 100 µM sodium citrate, 100 µM glucose, 100 µM caffeine and 100 µM DHA were added to a stirred solution containing phosphate buffer 0.1 M (pH 7). The inset shows the effect of the addition of 50 µM sodium ascorbate compared to the same concentration of hydrogen peroxide.
