# Supplementary material for: A Comparative Study of Poly(Azure A) Film-Modified Disposable Electrodes for Electrocatalytic Oxidation of H2O2: Effect of Doping Anion
Source: Polymers (Basel). 2018 Jan 6;10(1):48. doi: 10.3390/polym10010048 (PMC6414827; doi:10.3390/polym10010048)
Supplement: Supplementary file 1 [file polymers-10-00048-s001.docx]

**Supplementary Materials**

A comparative study of poly(azure A) film-modified disposable electrodes for electrocatalytic oxidation of H_2_O_2_: Effect of doping anion

Jerónimo Agrisuelas, María-Isabel González-Sánchez, Beatriz Gómez-Monedero and Edelmira Valero*





Figure S1. Polarization curves of 1 mg·mL^−1^ azure A in 0.02 M KCl (A) and KNO_3_ (B) aqueous solutions at scan rate 10 mV·s^−1^ between −0.25 V and 1 V. The voltammetry cycle stars at 0.5 V.





Figure S2. Calibration straight line of the PAA(DS) obtained from the amperometric response of such electrode upon successive additions of H_2_O_2_ in phosphate buffer solution (pH 7) at 0.5 V. The error bars correspond to standard deviations between three replicates using the same electrode.





Figure. S3. Amperometry of the influence of certain compounds measured at 0.5 V. 50 μM hydrogen peroxide, 100 μM ethanol, 100 μM sodium citrate, 100 μM glucose, 100 μM caffeine and 100 μM DHA were added to a stirred solution containing phosphate buffer 0.1 M (pH 7). The inset shows the effect of the addition of 50 μM sodium ascorbate compared to the same concentration of hydrogen peroxide.
